# Effect of COVID-19 on the characteristics and outcome of patients who have otitis media with effusion: a case-control study

**DOI:** 10.1186/s13005-024-00429-x

**Published:** 2024-05-10

**Authors:** Yue Fan, Wei Liu, Yinan Liang, Xin Xia, Fangxu Yan, Xingming Chen

**Affiliations:** 1 Department of Otolaryngology-Head and Neck Surgery, Peking Union Medical College, Peking Union Medical College Hospital, Chinese Academy of Medical Sciences, Beijing, China; 2 First Affiliated Hospital of Harbin Medical University, Harbin Medical University, Harbin, China

**Keywords:** COVID-19, Otitis media with effusion, Outcome, SARS-CoV-2

## Abstract

**Background:**

Few studies have examined the otologic symptoms of Coronavirus disease 2019 (COVID-19). The objective of this study was to identify the effect of COVID-19 on the characteristics and outcomes of patients who have otitis media with effusion (OME).

**Methods:**

This case-control study compared the characteristics and outcomes of OME patients who did or did not have COVID-19. A total of 65 patients with previous COVID-19 and 40 patients who did not have COVID-19 (controls) were enrolled from October 1, 2022 to January 31, 2023 at a single institution in China. Demographics, medical histories, morbidities, hearing test results, treatments, and outcomes of the two groups were compared.

**Results:**

The COVID-19 group had significantly better outcomes from OME than the control group, with higher rates of complete resolution (64.6% vs. 30%) and improvement (30.8% vs. 17.5%), and a lower rate of persistent OME (4.6% vs. 52.5%). Previous COVID-19 was independently associated with a more favorable OME outcome in three multivariate logistic regression models. The COVID-19 group also had a greater improvement in hearing threshold based on air-bone gap measurements.

**Conclusion:**

The outcomes of OME patients who had previous COVID-19 were generally good, in that most patients responded well to treatment and achieved complete resolution or improvement within one month.

## Introduction

Coronavirus disease 2019 (COVID-19) is a highly infectious respiratory illness whose main symptoms are fever, coughing, sore throat, fatigue, headache, muscle pain, and hemoptysis [1–3]. There has been a global effort to understand all of the clinical manifestations of COVID-19 in order to provide better preventive measures and treatments. COVID-19 can also cause a variety of ear, nose, and throat (ENT) symptoms, including nasal congestion, taste and smell disturbances, dysphagia, tinnitus, vertigo, and hearing loss. However, only a small number of studies have examined the otologic symptoms of COVID-19.

Some researchers concluded that because COVID-19 is mainly a respiratory tract disease, it has the potential to affect Eustachian tube function and middle ear mucosa, and could therefore lead to hearing loss [4]. Other studies reported detection of the severe acute respiratory syndrome coronavirus 2 (SARS-CoV-2) in the middle ear and middle ear fluid of necropsy samples, and suggested that otitis media should be considered a manifestation or symptom of COVID-19 [5–7]. We noticed an increase in the number of patients presenting at our department who had otitis media with effusion (OME) following the recent increase in the number of COVID-19 cases in China (December 2022).

OME is characterized by the presence of fluid in the tympanic cavity, without any overt symptoms or signs of systemic infection. Although OME is not considered life-threatening, it can cause significant hearing loss and long-term complications when ignored. For example, chronic OME can lead to adhesive otitis media, tympanosclerosis, and ossicular necrosis [8]. Therefore, further studies of the relationship of COVID-19 with OME are needed so that better outcomes can be provided to these patients.

We are unaware of any previous studies that performed audiological evaluations and assessed the effects of treatment and outcomes of patients who had COVID-19-related OME, and there is little is known about the specific factors that affect the risk for COVID-19-related OME. The objective of this study of patients with OME was to compare the clinical characteristics, including hearing function and outcomes, of patients with and without a history of COVID-19. The ultimate aim is to prevent the long-term consequences of OME from COVID-19, and to improve the quality of life in this patient population.

## Materials and methods

### Study population

This study was conducted in the Outpatient Department of Otorhinolaryngology, Head and Neck Surgery at the Peking Union Medical College Hospital (PUMCH) from October 1, 2022 to January 31, 2023. Ethical approval was obtained from the Institutional Review Board of PUMCH.

A total of 105 patients were included. All patients were diagnosed with OME based on physical examinations and auditory tests. The inclusion criteria were: (*i*) age of at least 18 years; (*ii*) presence of amber fluid behind tympanic membrane without signs of acute inflammation or tympanic membrane perforation in an otoscopic examination; (*iii*) pure tone audiometry (PTA) results revealing a mean air-bone gap (ABG) of more than 10 dB, calculated as the average of measurements at 500, 1000, 2000, and 4000 Hz; (*iv*) type B or C tympanometry results; and (*v*) receipt of follow-up for at least 60 days. Patients were excluded if they had previous tympanoplasty, mastoidectomy, or an identifiable cause for OME (such as nasopharyngeal carcinoma or nasal polyps).

Patients were classified into two groups according to COVID-19 status. The COVID-19 group (*n* = 65) consisted of patients who were previously positive for SARS-CoV-2 in a polymerase chain reaction (PCR) test or an antigen test from a nasopharyngeal swab, but tested negative for SARS-CoV-2 at the time of the OME examination. These patients developed aural symptoms within 21 days of the positive COVID-19 test result. Patients in the control group (*n* = 40) had no known previous positivity for SARS-CoV-2 in a PCR test, antibody test, or antigen test, and had no clinical signs suggesting COVID-19 during the pandemic (anosmia, ageusia, fever, dyspnea, cough, myalgia, headache).

### Follow-up and classification of outcomes

Follow-ups were conducted *via* outpatient visits and telephone. PTA and tympanometry tests were administered to confirm outcomes during visits at the outpatient clinic. The status of an affected ear was classified as “complete resolution”, “improvement”, or “persistence” according to strictly established criteria. “Complete resolution” was defined by one or more of following: (*i*) no aural symptoms of OME; (*ii*) mean ABG within 10 dB at 500, 1000, 2000, and 4000 Hz; or (*iii*) change to a type A tympanogram. “Improvement” was defined by one or more of the following: (*i*) significant relief from the aural symptoms of OME; (*ii*) 10 dB or greater decrease of the mean air conduction hearing threshold; (*iii*) change from a type B to type C tympanogram. “Persistence” was defined by one or more of the following: (*i*) no significant resolution of the aural symptoms of OME; (*ii*) 10 dB or smaller decrease of the mean the air conduction hearing threshold; (*iii*) persistence of a type B or type C tympanogram; or (*iv*) receipt of ventilation tube insertion (VTI).

### Statistical analysis

Continuous data were presented as means ± standard deviations (SDs) or as medians with interquartile ranges (IQRs), and categorical data as numbers and percentages. Variables were compared using a *t*-test (continuous variables) or a chi-square test (categorical variables). A one-way analysis of variance (ANOVA) with Tukey’s *post hoc* test was used for multiple comparisons. Univariate logistic regression analysis and the log-rank test were used to determine the significance of differences in qualitative and quantitative variables in the two groups. Adjusted ORs and 95% CIs were then calculated using multivariate analyses. Variables with a *P*-value below 0.1 in the univariate analysis were entered into the multivariate logistic analysis. Multivariate logistic regression analyses were used to identify variables that had independent and significant associations with adjustment for different confounding factors (three models). All tests were 2-sided, and a *P*-value below 0.05 was considered significant. All statistical analyses were performed using R software (http://www.R-project.org, The R Foundation, Version: 3.6.3, accessed on 12 February 2020).

## Results

The entire cohort of OME patients consisted of 53 women (50.5%) and 52 men (49.5%), the mean age was 50.5 ± 16.3 years, and the age range was 18 to 90 years (Table 1). Seventy-seven patients (73.3%) had one affected ear and 28 patients (26.7%) had two affected ears. We therefore performed an analysis of 133 affected ears. There were 65 patients (80 affected ears) in the COVID-19 group and 40 patients (53 affected ears) in the control group.

**Table 1:** Demographic and clinical characteristics of patients in the COVID-19 and control groups.*

	Total (*n* = 105)	Control (*n* = 40)	COVID-19 (*n* = 65)	*P* Value
**Sex**				0.259
Female	53 (50.5)	23 (57.5)	30 (46.2)	
Male	52 (49.5)	17 (42.5)	35 (53.8)	
**Age, years**	50.5 ± 16.3	51.3 ± 19.2	50.0 ± 14.4	0.703
**Affected ear**				0.57
Left	34 (32.4)	12 (30)	22 (33.8)	
Right	43 (41.0)	15 (37.5)	28 (43.1)	
Both	28 (26.7)	13 (32.5)	15 (23.1)	
**Duration of aural symptoms, days**	40.4 ± 59.9	79.3 ± 81.4	16.5 ± 16.4	**< 0.001**
**Specific aural symptoms**				
Otalgia				0.421
No	88 (83.8)	35 (87.5)	53 (81.5)	
Yes	17 (16.2)	5 (12.5)	12 (18.5)	
Aural fullness				**0.011**
No	39 (37.1)	21 (52.5)	18 (27.7)	
Yes	66 (62.9)	19 (47.5)	47 (72.3)	
Hearing loss				0.87
No	15 (14.3)	6 (15)	9 (13.8)	
Yes	90 (85.7)	34 (85)	56 (86.2)	
History of OME				0.071
No	64 (61.0)	20 (50)	44 (67.7)	
Yes	41 (39.0)	20 (50)	21 (32.3)	
History of tympanocentesis				**0.002**
No	80 (76.2)	24 (60)	56 (86.2)	
Yes	25 (23.8)	16 (40)	9 (13.8)	
History of VTI				**0.029**
No	92 (87.6)	31 (77.5)	61 (93.8)	
Yes	13 (12.4)	9 (22.5)	4 (6.2)	
**Comorbidities**				
Hypertension				0.87
No	90 (85.7)	34 (85)	56 (86.2)	
Yes	15 (14.3)	6 (15)	9 (13.8)	
Diabetes mellitus				1
No	99 (94.3)	38 (95)	61 (93.8)	
Yes	6 (5.7)	2 (5)	4 (6.2)	
Tumor				0.132
No	84 (80.0)	35 (87.5)	49 (75.4)	
Yes	21 (20.0)	5 (12.5)	16 (24.6)	

*Numbers indicate n (%) or mean ± SD.

OME, otitis media with effusion; VTI, ventilation tube insertion

### Characteristics of patients in the COVID-19 and control groups

The most common aural symptoms in the COVID-19 group were hearing loss (86.2%) and aural fullness (72.3%); significantly fewer patients in the control group reported aural fullness (47.5%, *P* = 0.011). Only 12 patients (18.5%) in the COVID-19 group and 5 patients (12.5%) in the control group reported transient earache (*P* = 0.421). The duration of aural symptoms before visiting the clinic was 16.5 ± 16.4 days in the COVID-19 group and 79.3 ± 81.4 days in the control group (*P* < 0.001).

Twenty-one patients (32.3%) in the COVID-19 group reported previous OME and complete resolution for more than 6 months. A greater percentage of patients in the control group had a history of OME (50%), but the difference was not significant (*P* = 0.071). The control group had significantly higher percentages of patients with previous of tympanocentesis (40.0% vs. 13.8%, *P* = 0.002) and VTI (22.5% vs. 6.2%, *P* = 0.029). The two groups had similar percentages of patients with hypertension (13.8% vs. 15.0% *P* = 0.87), diabetes mellitus (6.2% vs. 5.0%, *P* = 1.0), and uterine fibroids, breast cancer, bladder cancer, and other non-head and neck benign or malignant tumors (24.6% vs. 12.5%, *P* = 0.132).

We also analyzed the initial ABG and tympanometry results in 133 ears. The difference in the initial average ABG in the COVID-19 group (22.8 ± 8.4 dB, range: 10.0 to 47.5 dB) and the control group (25.8 ± 10.6 dB, range 10.0 to 53.8 dB) was only 2.9 ± 1.9 dB (*P* = 0.0784). Overall, 65 of 80 ears (81.25%) had a type B tympanogram, and the other 15 ears (18.75%) had a type C tympanogram in COVID-19 group.

### Treatments and outcomes of patients in the COVID-19 and control groups

The patients received various treatments for OME, mainly intranasal steroid, nasal decongestant, mucolytics (Table 2). A small number of patients were prescribed oral antibiotics, systemic steroids, or ear drops that contained a corticosteroid and an antibiotic. Significantly more patients in the COVID-19 group received a nasal decongestant (56.9% vs. 35%, *P* = 0.029), but the two groups did not differ in the other types of other conservative treatments. Significantly more patients in the control group received tympanocentesis (65% vs. 41.5%, *P* = 0.02).

**Table 2:** Treatments and outcomes of patients in the COVID-19 and control groups*

	Total (*n* = 105)	Control (*n* = 40)	COVID-19 (*n* = 65)	*P* Value
**Treatment**				
Intranasal steroid				0.608
No	40 (38.1)	14 (35)	26 (40)	
Yes	65 (61.9)	26 (65)	39 (60)	
Nasal decongestant				**0.029**
No	54 (51.4)	26 (65)	28 (43.1)	
Yes	51 (48.6)	14 (35)	37 (56.9)	
Mucolytic				0.281
No	41 (39.0)	13 (32.5)	28 (43.1)	
Yes	64 (61.0)	27 (67.5)	37 (56.9)	
Oral antibiotic				0.325
No	90 (85.7)	36 (90)	54 (83.1)	
Yes	15 (14.3)	4 (10)	11 (16.9)	
Oral steroid				0.404
No	99 (94.3)	39 (97.5)	60 (92.3)	
Yes	6 (5.7)	1 (2.5)	5 (7.7)	
Topical steroid				0.295
No	101 (96.2)	40 (100)	61 (93.8)	
Yes	4 (3.8)	0 (0)	4 (6.2)	
Topical antibiotic				1
No	100 (95.2)	38 (95)	62 (95.4)	
Yes	5 (4.8)	2 (5)	3 (4.6)	
Tympanocentesis				**0.02**
No	52 (49.5)	14 (35)	38 (58.5)	
Yes	53 (50.5)	26 (65)	27 (41.5)	
**Outcome**				**< 0.001**
Complete resolution	54 (51.4)	12 (30)	42 (64.6)	
Improvement	27 (25.7)	7 (17.5)	20 (30.8)	
Persistence	24 (22.9)	21 (52.5)	3 (4.6)	

*Numbers indicate n (%)

At the time of the follow-up assessment (62 to 165 days following the first visit), the COVID-19 group had 42 patients (64.6%) who had complete resolution, 20 (30.8%) who had improvement, and only 3 (4.6%) who had persistent OME. In contrast, the control group had 21 patients (52.5%) with persistent OME. The COVID-19 group had a significantly better outcome than the control group (*P* < 0.001).

### Factors associated with OME outcome

We then performed a logistic regression analysis to investigate the relationship between different variables and OME outcome, in which “complete resolution” and “improvement” were considered as good outcomes, and “persistence” was considered a poor outcome (Table 3). The univariate (unadjusted) logistic regression analysis indicated that outcome was associated with previous COVID-19, duration of aural symptoms, history of OME, history of tympanocentesis, and history of VTI.

**Table 3:** Univariate logistic regression analysis of the association of different variables with OME outcome of complete resolution or improvement

Variable	OR (95% CI)	*P* value
COVID-19 positive	0.04 (0.01, 0.16)	< 0.001
Sex	1.27 (0.51, 3.17)	0.605
Age	1.03 (1, 1.06)	0.09
Affected ear		
Left	Reference	
Right	1.99 (0.62, 6.43)	0.248
Bilateral	2.32 (0.66, 8.13)	0.188
Duration of aural symptoms	1.02 (1.01, 1.03)	**0.001**
History of OME	3.53 (1.36, 9.11)	**0.009**
History of tympanocentesis	4.05 (1.51, 10.88)	**0.006**
History of VTI	7.6 (2.2, 26.28)	**0.001**
HT	1.27 (0.37, 4.43)	0.705
DM	0.66 (0.07, 5.95)	0.712
Tumors	0.3 (0.06, 1.38)	0.121
Intranasal steroid	1.31 (0.5, 3.41)	0.585
Nasal decongestant	0.44 (0.17, 1.15)	0.093
Mucolytics	1.38 (0.53, 3.58)	0.514
Oral antibiotics	0.48 (0.1, 2.27)	0.352
Tympanocentesis	2.38 (0.92, 6.18)	0.075

We then performed multivariate analyses using three different models that adjusted for different confounding factors (Table 4). The significance of the relationship between previous COVID-19 and OME remained after adjustment for age and sex (Model I), duration of aural symptoms, history of OME, history of tympanocentesis, and history of VTI (Model II), and all six of these variables (Model III).

**Table 4:** Multivariate logistic regression analysis of variables associated with OME outcome of complete resolution or improvement in models that adjusted for different confounders*

	Unadjusted OR (95%CI)	*P* value	Model I aOR (95%CI)	*P* value	Model II aOR (95%CI)	*P* value	Model III aOR (95%CI)	*P* value	
COVID-19 (−)	Reference		Reference		Reference		Reference		
COVID-19 (+)	0.04 (0.01,0.16)	**< 0.001**	0.04 (0.01,0.15)	**< 0.001**	0.07 (0.02,0.31)	**< 0.001**	0.08 (0.02,0.35)	**0.001**	

*Model I adjusted for age and sex; Model II adjusted for duration of aural symptoms, history of OME, history of tympanocentesis, and history of VTI; Model III adjusted for all Model I and Model II variables

We analyzed the recovery times in the 62 patients in COVID-19 group who had good outcomes (complete resolution or improvement; Table 5). For this analysis, we classified the recovery time as less than 2 weeks, 2 to 4 weeks, 4 to 8 weeks, and more than 8 weeks. Twenty-one patients (33.9%) had relief of symptoms within 2 weeks and 30 patients (48.4%) had relief of symptoms in 2 to 4 weeks. The duration of aural symptoms before the initial presentation was associated with the time needed for recovery after initial presentations (*P* = 0.025). None of the other analyzed parameters were significantly associated with recovery time.

**Table 5:** Relationship of patient characteristics with time needed for OME outcome of complete resolution or improvement.*

	≤ 2 weeks (*n* = 21)	2 to 4 weeks (*n* = 30)	4 to 8 weeks (*n* = 7)	> 8 weeks (*n* = 4)	*P* Value
Sex					0.144
Female	9 (42.9)	17 (56.7)	1 (14.3)	3 (75)	
Male	12 (57.1)	13 (43.3)	6 (85.7)	1 (25)	
Mean age, years	51.6 ± 14.4	48.6 ± 14.2	47.4 ± 14.6	49.2 ± 14.4	0.865
Affected ear					0.666
Left	6 (28.6)	11 (36.7)	1 (14.3)	3 (75)	
Right	9 (42.9)	13 (43.3)	4 (57.1)	1 (25)	
Bilateral	6 (28.6)	6 (20)	2 (28.6)	0 (0)	
Duration of aural symptoms, days	15.6 ± 13.1	15.7 ± 13.9	13.1 ± 8.2	41.0 ± 41.1	**0.025**
Aural symptoms after onset, days	7.8 ± 5.6	10.6 ± 6.8	7.6 ± 5.5	9.8 ± 9.2	0.411
Previous OME					0.655
No	16 (76.2)	19 (63.3)	5 (71.4)	2 (50)	
Yes	5 (23.8)	11 (36.7)	2 (28.6)	2 (50)	
Previous tympanocentesis					0.142
No	19 (90.5)	25 (83.3)	7 (100)	2 (50)	
Yes	2 (9.5)	5 (16.7)	0 (0)	2 (50)	
Previous VTI					1
No	19 (90.5)	28 (93.3)	7 (100)	4 (100)	
Yes	2 (9.5)	2 (6.7)	0 (0)	0 (0)	
Hypertension					0.535
No	19 (90.5)	26 (86.7)	5 (71.4)	4 (100)	
Yes	2 (9.5)	4 (13.3)	2 (28.6)	0 (0)	
Diabetes mellitus					0.135
No	18 (85.7)	30 (100)	7 (100)	4 (100)	
Yes	3 (14.3)	0 (0)	0 (0)	0 (0)	
Tumor					0.804
No	14 (66.7)	23 (76.7)	6 (85.7)	3 (75)	
Yes	7 (33.3)	7 (23.3)	1 (14.3)	1 (25)	
Tympanocentesis procedures					0.108
0	11 (52.4)	19 (63.3)	4 (57.1)	2 (50)	
1	10 (47.6)	9 (30)	1 (14.3)	1 (25)	
2	0 (0)	1 (3.3)	2 (28.6)	0 (0)	
3	0 (0)	1 (3.3)	0 (0)	1 (25)	

*Numbers indicate n (%) or mean ± SD

**Fig. 1:**
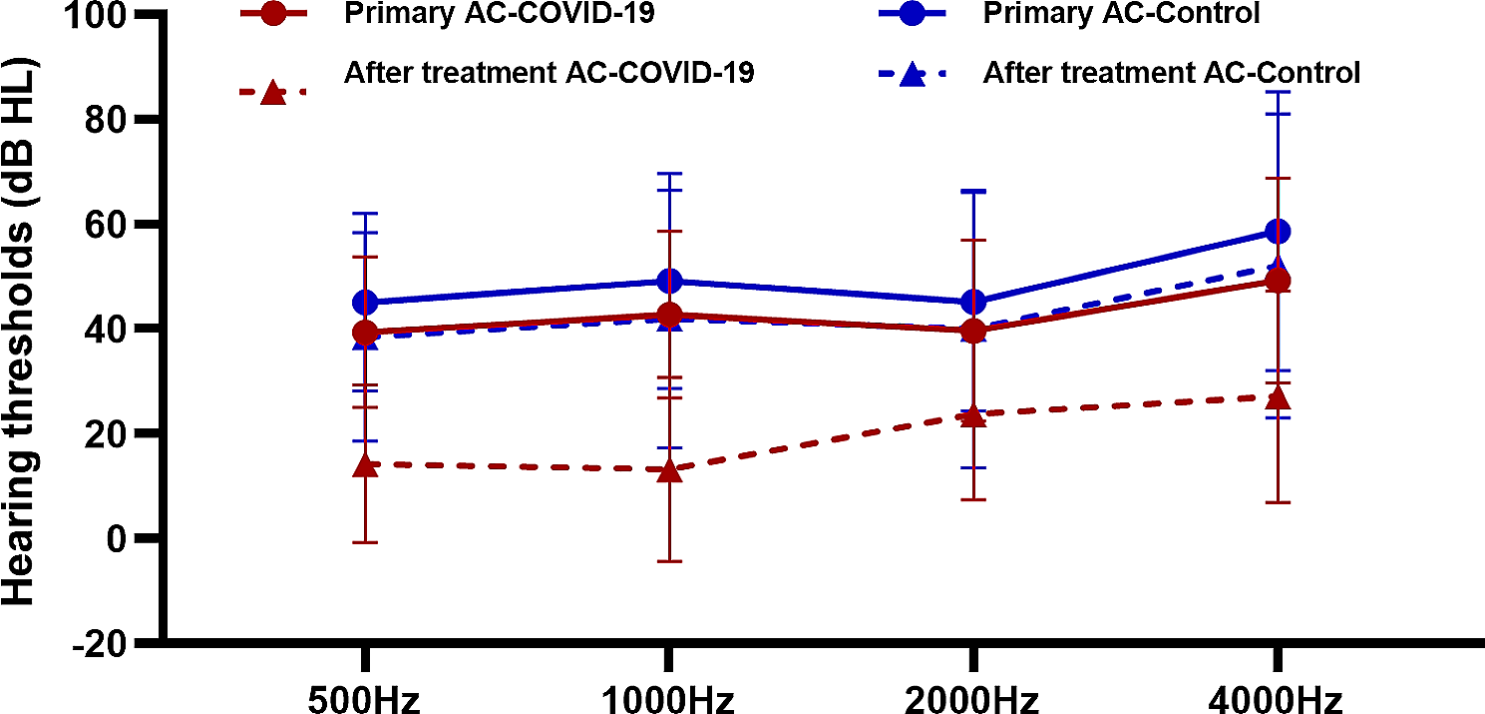
Hearing thresholds at four frequencies before and after treatment in the COVID-19 and control groups

We used PTA and tympanography to assess the hearing thresholds at the primary clinic visit and the last visit after treatment in 45 of 65 patients (69.2%) in the COVID-19 group and 29 of 40 patients (72.5%) in the control group (Fig. 1). These data were for 74 patients and 96 ears. After treatment, the average ABG decreased to 10.5 ± 9.3 dB (range: 0–36.3 dB) in the COVID-19 group and decreased to 19.6 ± 12.2 dB (range: 0–43.8 dB) in the control group (*P* < 0.0001). Thus, the COVID-19 group had a significantly greater improvement in hearing threshold.

## Discussion

Previous studies showed that viral infection of the middle ear viral could contribute to otologic pathology and symptoms such as otitis media and conductive hearing loss [9]. Fidan reported the first case of otitis media in a COVID-19 patient in April 2020 [10], and subsequent studies tentatively confirmed a relationship between COVID-19 and OME. For example, Raad et al. reported eight COVID-19 patients who had otitis media and no previous history of middle ear infections, and that one of these patients was positive for SARS-CoV-2 in a sample of middle ear fluid [5]. These studies thus suggest a causal relationship between COVID-19 and OME, and that OME should be considered a possible symptom of COVID-19. Our data are from a busy otologic practice in Beijing. We noticed an increase in the number of patients presenting with OME during the same period when there was an increase in the number of COVID-19 patients in the general population.

Most of our patients complained of hearing loss as the main symptom, and this was followed by the symptom of aural fullness. Only a small number of our OME patients experienced transient earache, in contrast to previous studies. In particular, Sebothoma et al. reviewed eight studies that examined the effects of COVID-19 on middle ear function and found that middle ear-related symptoms, including otalgia and aural fullness, were common. In fact, among all symptoms, otalgia was the most common middle ear-related symptom reported in these eight studies [11]. This discrepancy may be related to differences in the sample sizes and patient ages.

The duration of ear symptoms was significantly shorter in our COVID-19 group than in our control group, possibly because the COVID-19 patients were more alert to symptoms and sought earlier medical attention. Our two groups had no significant differences in comorbidities; hypertension, diabetes mellitus, and tumors were the most common comorbidities in both groups.

Several studies have documented the effect of COVID-19 on hearing. For example, a systematic review and meta-analysis of audiovisual symptoms in COVID-19 patients reported the prevalence of hearing loss was 7.6% [12]. Another meta-analysis of 12 publications demonstrated that the event rate of hearing loss was 3.1% in patients with confirmed COVID-19 [13]. In this later study, COVID-19 patients with OME presented with moderate conductive hearing loss, and most of the hearing loss was restorable.

To further understand the effect that COVID-19 on the auditory system, the potential long-term effects, and the effect of different treatments, we performed a follow up of patients who received different treatments and recorded their long-term outcomes. Our results showed that the outcome of the COVID-19 group was significantly better than that of the control group, in that 95.4% of the COVID-19 patients achieved symptom improvement or complete recovery and 82.3% of these patients experienced this outcome within 1 month. Patients who had longer recovery times (more than 8 weeks) had a significantly longer duration of symptoms prior to the first clinical visit (*P* = 0.025), suggesting that early diagnosis and treatment might have contributed to faster recovery. Previous infection by SARS-CoV-2, duration of aural symptoms prior to initial presentation, history of OME, history of tympanocentesis, and history of VTI were all associated with OME outcome in the univariate logistic regression analysis. And after adjustment for confounders in three multivariate models, COVID-19 was remained independently associated OME outcome.

For conservative treatments, most patients were prescribed intranasal steroids, nasal decongestants, and mucolytics (alone or in combination). The American Academy of Otolaryngology-Head and Neck Surgery 2016 Clinical Practice Guidelines showed no significant benefit of using oral or intranasal steroids for treatment of OME [14]. Despite this recommendation, some clinicians still use these treatments for patients with OME. In the present study, a small number of patients were prescribed oral and topical corticosteroids. None of the medications had a significant effect on OME outcome.

Conservative treatments are usually preferred for patients with newly diagnosed OME. Invasive treatments, such as tympanocentesis or VTI, are effective options that are usually performed when conservative treatments fail [15]. In our COVID-19 group, 27 patients (41%) received a tympanocentesis, and 6 patients (9.2%) received more than one aspiration. Many patients underwent tympanocentesis at the initial visit, probably to restore hearing as quickly as possible, or because an insufficient understanding of a patient’s condition led to more aggressive treatment. However, our results showed that tympanocentesis had no significant effect on OME outcome. This suggests that less invasive treatments may be preferable for COVID-19-associated OME.

## Conclusion

COVID-19-related OME can cause moderate conductive hearing loss. Our results showed that outcome from OME had significant relationships with the duration of ear symptoms before initial presentation and with previous COVID-19. The outcomes of patients with COVID-19-related OME were generally good, in that most patients achieved improvement within 1 month. Our results suggest that early diagnosis and appropriate treatment may reduce the long-term consequences of OME in patients with previous COVID-19.

## Data Availability

No datasets were generated or analysed during the current study.

## Abbreviations

ABG: Air-bone gap
ANOVA: A one-way analysis of variance
COVID-19: Coronavirus disease 2019
ENT: Ear, nose, and throat
IQRs: Interquartile ranges
OME: Otitis media with effusion
PCR: Polymerase chain reaction
PTA: Pure tone audiometry
SARS-CoV-2: Severe acute respiratory syndrome coronavirus 2
SDs: Standard deviations
VTI: Ventilation tube insertion
